# c-Myc suppresses miR-451⊣YWTAZ/AKT axis via recruiting HDAC3 in acute myeloid leukemia

**DOI:** 10.18632/oncotarget.12679

**Published:** 2016-10-15

**Authors:** Rui Su, Jia-Nan Gong, Ming-Tai Chen, Li Song, Chao Shen, Xin-Hua Zhang, Xiao-Lin Yin, Hong-Mei Ning, Bing Liu, Fang Wang, Yan-Ni Ma, Hua-Lu Zhao, Jia Yu, Jun-Wu Zhang

**Affiliations:** ^1^ The State Key Laboratory of Medical Molecular Biology, Department of Biochemistry and Molecular Biology, Institute of Basic Medical Sciences, Chinese Academy of Medical Sciences and Peking Union Medical College, Beijing, China; ^2^ Department of Hematology, The 303 Hospital, Nanning, Guangxi, China; ^3^ Department of Hematopoietic Stem Cell Transplantation, Affiliated Hospital to Academy of Military Medical Sciences, The 307 Hospital, Beijing, China; ^4^ State Key Laboratory of Proteomics, Translational Medicine Center of Stem Cells, 307-lvy Translational Medicine Center, Laboratory of Oncology, Affiliated Hospital of Academy of Military Medical Sciences, Beijing, China

**Keywords:** acute myeloid leukemia, microRNA-451, c-Myc, HDAC3, YWHAZ

## Abstract

Aberrant activation of c-Myc plays an important oncogenic role via regulating a series of coding and non-coding genes in acute myeloid leukemia (AML). Histone deacetylases (HDACs) can remove acetyl group from histone and regulate gene expression via changing chromatin structure. Here, we found miR-451 is abnormally down-regulated in AML patient samples; c-Myc recruits HDAC3 to form a transcriptional suppressor complex, co-localizes on the *miR-451* promoter, epigenetically inhibits its transcription and finally induces its downregulation in AML. Furthermore, our *in vitro* and *in vivo* results suggest that miR-451 functions as a tumor suppressor via promoting apoptosis and suppressing malignant cell proliferation. The mechanistic study demonstrated that miR-451 directly targets *YWHAZ* mRNA and suppresses YWHAZ/AKT signaling in AML. Knockdown of *c-Myc* results in restoration of miR-451 and inhibition of YWHAZ/AKT signaling. In AML patients, low level of miR-451 is negatively correlated with high levels of c-Myc and YWHAZ, while c-Myc level is positively related to YWHAZ expression. These results suggested that c-Myc⊣miR-451⊣YWHAZ/AKT cascade might play a crucial role during leukemogenesis, and reintroduction of miR-451 could be as a potential strategy for AML therapy.

## INTRODUCTION

Acute myeloid leukemia (AML) is a heterogenous disorder of aggressive hematopoietic disease characterized by malignant proliferation of clonal neoplastic cells and differentiation arrest of myeloid blasts [1–3]. It is widely reported that Notch signaling [4], PI3K/AKT pathway [5], JUN and c-Myc are indispensable and critical for leukemiagenesis [6–9]. AML-associated fusion proteins, such as AML-ETO and PML/RARA, could induce expression of *c-Myc* [10]. Hyperactivation of *c-Myc* is one of the most frequent events associated with AML and c-Myc is essential for cell growth, hepatopoiesis differentiation and leukemogenesis [11]. Histone deacetylases (HDACs) are involved in remodeling nucleosomes and chromatin via removing acetyl group from histone and function as critical transcriptional co-repressors in epigenetic regulation of gene expression [12, 13]. Interestingly, HDAC families are abnormally up-regulated in leukemia [14] and HDAC inhibitors have been used to treat malignant leukemia in clinical studies [15–17]. However, the synergetic regulation of c-Myc and HDAC is not clearly studied in AML.

MicroRNAs (miRNAs), a class of small noncoding RNA, are emerging as important posttranscriptional players during normal hematopoiesis and deregulation of specific miRNAs are associated with initiation, progression, diagnosis as well as prognosis of leukemia [18–20]. As hematopoietic differentiation, miR-451 is required for erythroid homeostasis and plays a crucial role in promoting erythroblast maturation [21, 22]. MiR-451 also functions as a tumor suppressor via targeting *c-Myc* mRNA in lung adenocarcinoma [23], *IKK-β* mRNA in hepatocellular carcinoma [24], *14-3-3ζ* mRNA in breast cancer [25]. However the expression level, the potential function of miR-451 and its correlation with c-Myc and HDAC family is not clear yet in AML.

Here, we found that hyperactivation of c-Myc could recruit HDAC3 to bind on the promoter region of *miR-451* and induce the deacethylation of histone, which finally results in deregulation of *miR-451* in AML. Moreover, *in vitro* and *in vivo* reintroduction of miR-451 could promote cell apoptosis and inhibit uncontrolled proliferation via directly targeting YWHAZ/ AKT signaling.

## RESULTS

### miR-451 is abnormally down-regulated in AML patients

**Figure 1:**
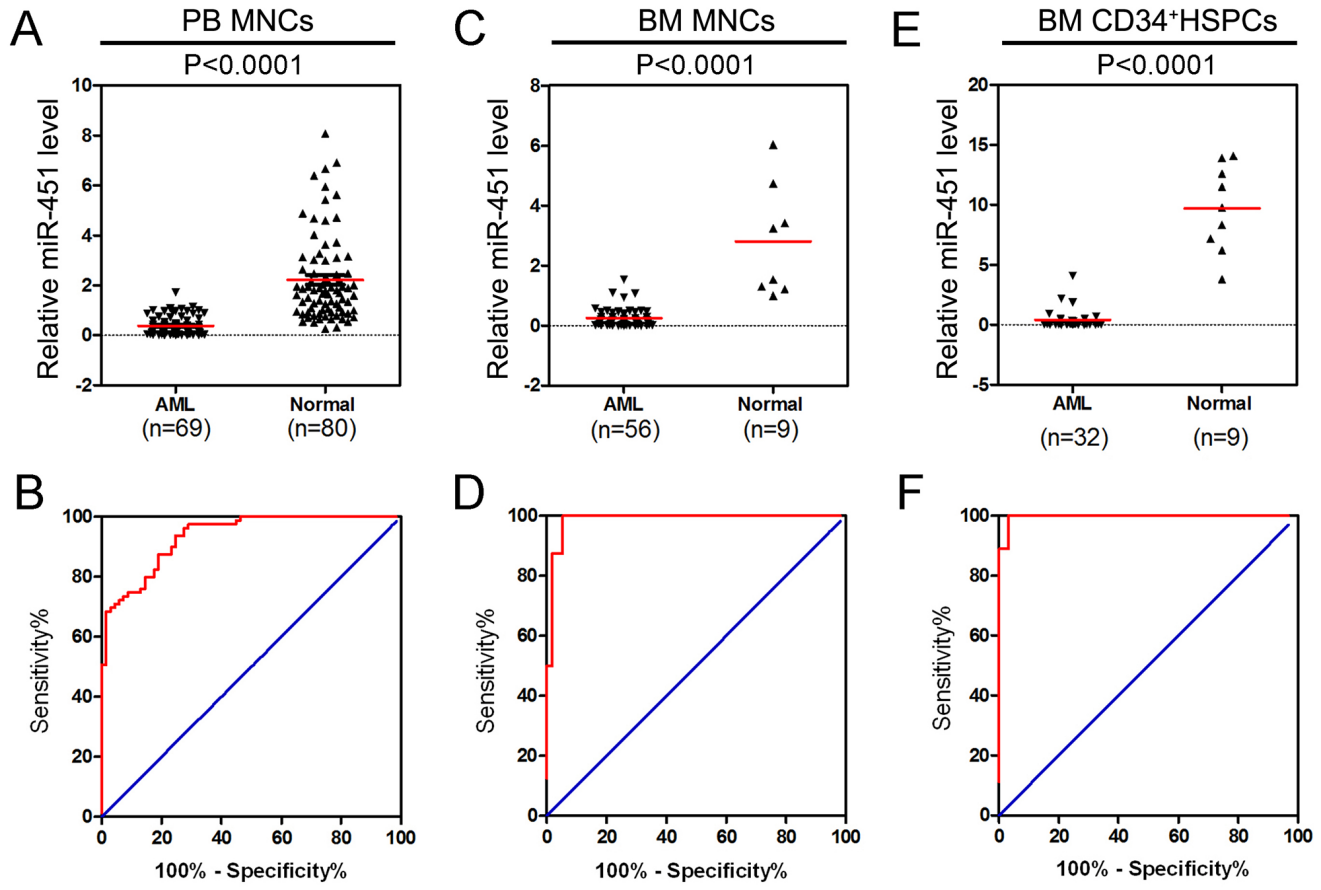
miR-451 is abnormally down-regulated in AML patients **A.** The expression of miR-451 was determined by stem-loop real-time-PCR in PB MNCs derived from 69 AML patients and 80 healthy donors. U6 snRNA was used as the internal control and each real-time PCR assay was performed in triplicate. **B.** ROC curve analysis of miR-451 expression in the PB MNC samples. The area under ROC curve (AUC), sensitivity and specificity were 93.5%, 98.7% and 98.5%, respectively. **C.** The relative level of miR-451 in BM MNCs from 56 AML patients and 9 healthy donors. **D.** ROC curve analysis of miR-451 expression in the BM MNC samples. The AUC, sensitivity and specificity were 98.7%, 87.5% and 98.2%, respectively. **E.** The relative expression of miR-451 in BM CD34^+^ HSPCs from 38 AML patients and 9 normal controls. **F.** ROC curve analysis of miR-451 expression in CD34^+^ HSPCs. The AUC, sensitivity and specificity were 99.7%, 88.9% and 96.7%, respectively.

We analyzed relative expression of miR-451 in peripheral blood (PB) mononuclear cells (MNCs) derived from 69 primarily diagnosed AML patients with different mutation and genomic translocation (Supplementary Table S1) and 80 healthy donors. The data indicated that miR-451 is abnormally down-regulated in the AML patients (Figure 1A). The receiver-operating characteristic (ROC) curve result suggested that expression level of miR-451 could be as a marker with high sensitivity and specificity for AML diagnosis (Figure 1B). Similarly, as shown in Figure 1C and 1E, the relative expression of miR-451 was also significantly suppressed in bone marrow (BM) MNCs and BM CD34^+^ hematopoietic stem/progenitor cells (HSPCs) of AML patients. Moreover, the ROC curves indicated that miR-451 level could also be as important marker for AML diagnosis in BM MNC samples and CD34^+^ HSPCs samples (Figure 1D and 1F). We did not observe significant difference of miR-451 expression among the AML FAB subtypes or cytogenetically normal AML (CN-AML) *vs* cytogenetically abnormal AML (CA-AML) (Supplementary Figure S1A–S1D). These results suggest miR-451 might function as a tumor suppressor in AML development.

### c-Myc directly suppresses *miR-451* expression by recruiting HDAC3 in AML

**Figure 2:**
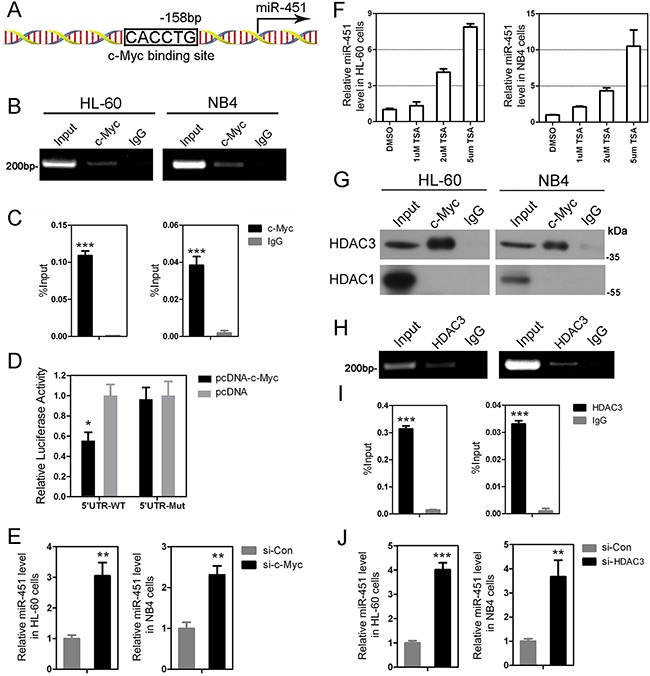
c-Myc suppresses expression of *miR-451* via recruiting HDAC3 **A.** A sketch showing the c-Myc binding site at −158 bp upstream of the *miR-451* promoter. The other potential binding sites, which are not been confirmed by ChIP-PCR, are not shown. **B.** The ChIP-PCR data indicate that c-Myc binds on the c-Myc binding site at −158 bp upstream of the *miR-451* promoter in HL-60 and NB4 cells. **C.** Quantitative ChIP-PCR demonstrates a significant enrichment of c-Myc on the promoter region of *miR-451* in the AML cells. **D.** Enforced expression of c-Myc by pcDNA-c-Myc tensfection inhibits the activity of *miR-451* promoter. The dual-luciferase reporter assay was performed in triplicate in HEK-293 cells. Error bars represent SD. * *P*<0.05; Student's t-test. **E.** Knowdown of *c-Myc* by si-c-Myc transfection increased miR-451 level in HL-60 and NB4 cells. **P<0.01. **F.** TSA treatment significantly induces expression of miR-451 in the AML cells at a dose-dependent manner. **G.** The Co-IP assay indicates that c-Myc can interact with HDAC3, but not HDAC1, in Hl-60 and NB4 cells. **H.** ChIP-PCR shows HDAC3 binds on the same location of *miR-451* promoter with c-Myc. **I.** Quantitative ChIP-PCR analysis demonstrated a significant enrichment of HDAC3 on the promoter region of *miR-451* in the AML cells. ***P<0.001. **J.** Inhibition of HDAC3 by si-HDAC3 transfection dramatically accelerates expression of miR-451 in the AML cells. **P<0.01; ***P<0.001.

To understand how *miR-451* is downregulated in AML patients, we used bioinformatics to analyze 2500 bp upstream of *miR-451* to see whether it contains potential binding sites for transcriptional factors that are involved in leukemogenesis. Interestingly, we identified multiple nonconsensus E box elements, which may be involved in potential binding sites of some transcriptional factors including c-Myc, on the *miR-451* promoter region. Our chromatin Immunoprecipitation (ChIP)-PCR showed that c-Myc could bind to one of these potential binding sites, which is located at −158 bp upstream of miR-451 (Figure 2A), in both NB4 and HL-60 AML cells (Figure 2B and 2C). To evaluate the effect of c-Myc activity on miR-451 expression, we constructed miR-451 promoter-reporter vectors containing wild-type or deleted c-Myc binding site for dual-luciferase reporter assay. The data indicated that forced expression of *c-Myc* could significantly inhibit the transcriptional activity of *miR-451* promoter, while deletion of the c-Myc binding site abolished this suppression in HEK-293T cells (Figure 2D). Moreover, knockdown of *c-Myc* in both NB4 and HL-60 cell lines by siRNAs transfection could promoted expression of miR-451 (Figure 2E). Our results suggested that c-Myc directly binds on the *miR-451* promoter and suppresses its expression, which may be one of the most important reasons which result in its deregulation in AML.

As reported, c-Myc could recruit HDAC1 or HDAC3 to suppress expression of specific genes, such as *HPP1* [26], *miR-29* [27], *miR-15/16-1* [28], which leads us to investigate whether the recruitment of HDAC1/3 is involved in the transcriptional repression of *miR-451* by c-Myc. To address this hypothesis, we firstly examined effects of deacetylase inhibitor Trichostatin A (TSA) on miR-451 expression. As shown in Figure 2F, TSA caused a dose-dependent increase of miR-451 expression in NB4 and HL-60 AML cells, which suggested the role of HDACs in miR-451 expression. Our Co-immunoprecipitation (Co-IP) results indicated that HDAC3, but not HDAC1, physiologically interacted with c-Myc in the AML cells (Figure 2G). Importantly, ChIP-PCR showed that HDAC3 could co-localize with c-Myc to the same region on the *miR-451* promoter (Figure 2H and 2I). And knockdown of *HDAC3* by siRNAs could increase miR-451 expression in NB4 and HL-60 AML cells (Figure 2J).

Taken together, our mechanistic data suggest that c-Myc recruits HDAC3 to co-localize on the *miR-451* promoter region, and repress its expression in AML.

### Forced expression of miR-451 decreases cell proliferation and increases cell apoptosis in AML

**Figure 3:**
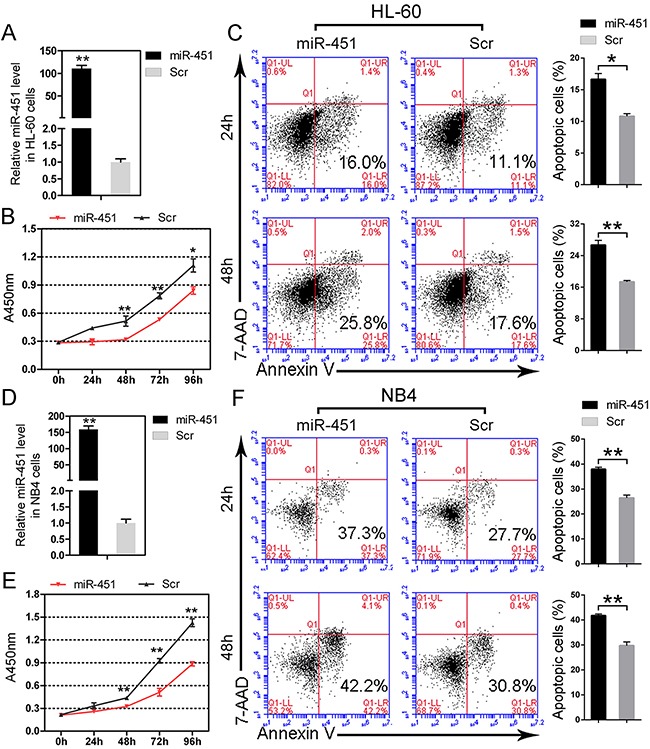
miR-451 restricts proliferation and induces apoptosis in AML cells **A.** The over-presence of miR-451 was confirmed by qPCR in HL-60 cells transfected with miR-451 mimics. **B.** Enforced expression of miR-451 inhibits proliferation of HL-60 cells. **C.** Enforced expression of miR-451 accelerates serum deprivation-induced apoptosis in HL-60 cells. A representative experiment is presented on the left and a statistical analysis of three independent experiments on the right. Error bars represent SD. *P<0.05; **P<0.01; Student's t-test. **D.** The over-presence of miR-451 was confirmed by qPCR in NB4 cells transfected with miR-451 mimics. **E.** Ectopic expression of miR-451 suppresses cell proliferation in NB4 cells. **F.** Overexpression of miR-451 promotes apoptosis in NB4 cells. A representative experiment is presented on the left and a statistical analysis of three independent experiments on the right. **P<0.01.

The above results show that c-Myc negatively regulates *miR-451* via recruiting HDAC3 on the promoter region of *miR-451* and results in its deregulation in AML. We then focused on the function of miR-451 in AML. To this aim, we first transfected miR-451 mimics into HL-60 and NB4 cells and detected its effect on cell proliferation and apoptosis. The over-presence of miR-451 in the HL-60 cells transfected with miR-451 mimics was confirmed (Figure 3A) by qPCR. Enforced expression of miR-451 significantly suppressed cell proliferation (Figure 3B) and induced cell apoptosis (Figure 3C) in the HL-60 cells. Similarly, forced expression of miR-451 (Figure 3D) also restrained cell growth (Figure 3E) and promoted cell apoptosis (Figure 3F) in the NB4 cells. While forced expression of miR-451 has little effects on cell cycle in AML cells (data not shown).

### Reintroduction of miR-451 significantly inhibits engraftment of leukemic cells and accelerates cell apoptosis *in vivo*

**Figure 4:**
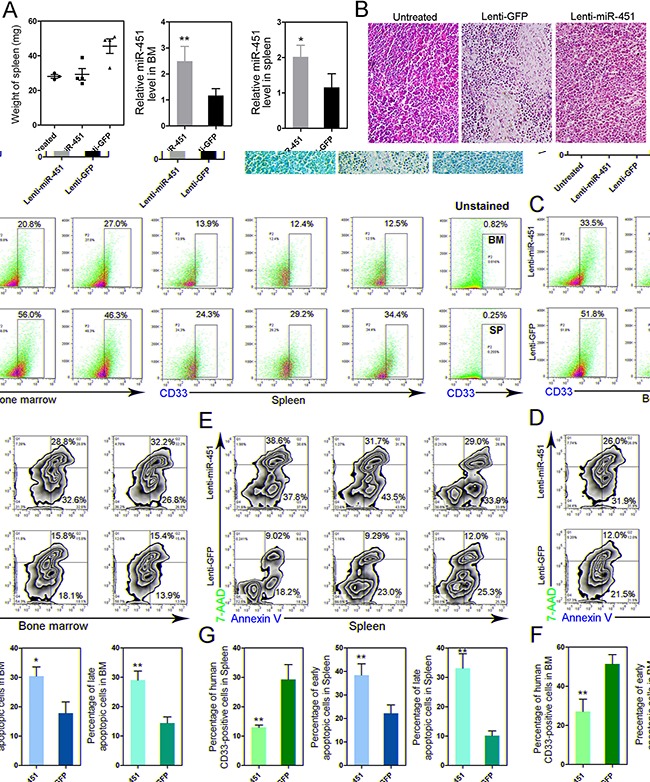
Reintroduction of miR-451 inhibits leukemic spreading and induces apoptosis in AML mouse model **A.** Enforced expression of miR-451 relieves splenomegaly induced by leukemia *in vivo* (left panel). Overexpression of miR-451 was confirmed by qRT-PCR in BM (middle panel) and spleen (right panel). **B.** H & E staining of spleen from indicated groups. **C.** Enforced expression of miR-451 suppressed engraftment of leukemic cells into BM and spleen. The human-specific CD33 surface marker was detected by flow cytometry to identify the human leukemia cells. **D** and **E.** Reintroduction of miR-451 induces cell apoptosis in BM (D) and spleen (E). **F** and **G.** The statistical results of flow cytometry data, which strongly suggest that reintroduction of miR-451 inhibits engraftment of leukemic cells and induces apoptosis *in vivo*. *P<0.05; **P<0.01, Student's t-test.

To further understand the anti-leukemia function of miR-451 and examine the potential possibility that reintroduction of miR-451 for AML treatment, we constructed the AML murine model via injection NB4 cells into NOD/SCID mice for *in vivo* study. As compared to the control group injected with Lenti-GFP, reintroduction of miR-451 mediated by Lenti-miR-451 injection significantly inhibited splenomegaly induced by leukemia in AML NOD/SCID mice (Figure 4A, left). The infection efficiency and enforced expression of miR-451 were confirmed in BM and spleen samples (Figure 4A, middle and right panel; Supplementary Figure S2A-S2C). Hematoxylin and eosin (H & E) staining showed that neoplastic infiltration was suppressed by Lenti-miR-451 in spleen (Figure 4B). More importantly, flow cytometry results strongly demonstrated that reintroduction of miR-451 dramatically inhibited engraftment of leukemic cells into BM (Figure 4C; Figure 4F, left panel) and spleens (Figure 4C; Figure 4G, left panel). Forced expression of miR-451 also induced apoptosis both in BM (Figures 4D; Figure 4F, middle and right panels) and spleens (Figures 4E; Figure 4G, middle and right panels).

Totally, our *in vivo* data also suggest miR-451 functions as a tumor suppressor via regulating cell proliferation and apoptosis, and reintroduction of miR-451 could partially release leukemic symptoms in AML.

### miR-451 directly suppresses YWHAZ-AKT signaling in AML cells

**Figure 5:**
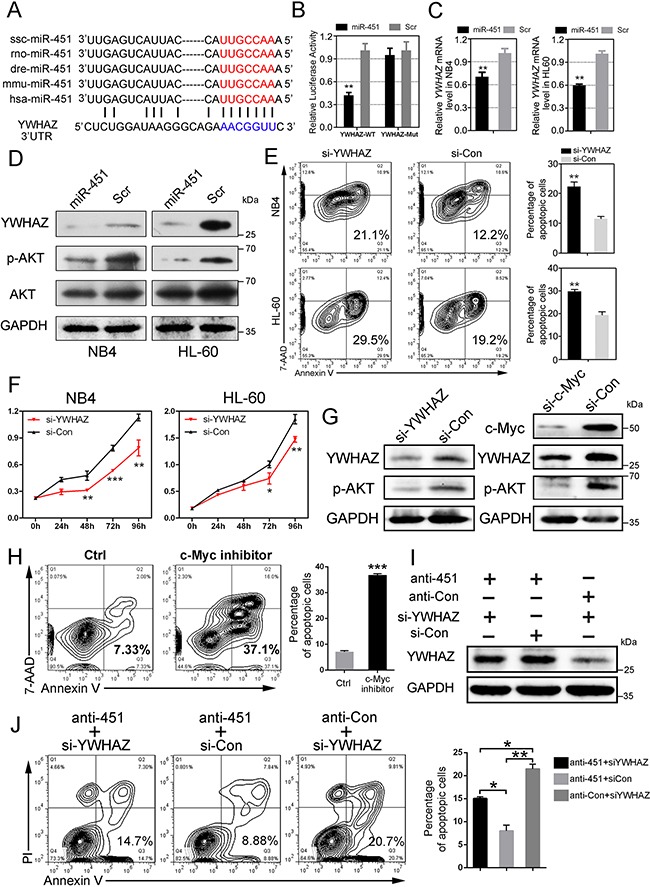
miR-451 directly targets *YWHAZ* and subsequently suppresses YWHAZ-AKT signaling in AML cells **A.** The predicated potential binding site of miR-451 on the 3'UTR of *YWHAZ*. The “seed sequence” of miR-451 is marked with red color. **B.** Enforced miR-451 expression inhibited relative luciferase activity of the construct containing the wild type *YWHAZ* 3'UTR, but not the mutant. The dual-luciferase reporter assay was performed in triplicate in HEK-293 cells. Error bars represent SD (n=3); **P<0.01; Student's t-test. **C.** Overexpression of miR-451 reduced *YWHAZ* mRNA level in NB4 and HL-60 cells. **P<0.01. **D.** Enforced miR-451 expression reduced YWHAZ and subsequently decreased p-AKT protein levels in NB4 and HL-60 cells. **E.** knockdown of *YWHAZ* promoted apoptosis of NB4 and HL-60 cells. A representative experiment is presented on the left and a statistical analysis of three independent experiments on the right. **P<0.01. **F.** Knockdown of *YWHAZ* restrains proliferation of NB4 and HL-60 cells. **G.** Either knockdown of Y*WHAZ* or knockdown of *c-Myc* inhibited activity of YWHAZ/AKT signaling in the AML cells. **H.** c-Myc inhibitor (JQ-1) treatment accelerated apoptosis in NB4 AML cells. A representative experiment is presented on the left and a statistical analysis of three independent experiments on the right. ***P<0.001. **I.** Immunoblotting of YWHAZ in the NB4 cells that were transfected with anti-451 (or anti-Con) for 24 h and then treated for another 48 h with si-YWHAZ (or si-Con). **J.** The cell apoptosis inhibition by anti-451 transfection can be rescued by re-transfection with si-YWHAZ. A representative experiment is presented on the left and a statistical analysis of three independent experiments on the right. *P<0.05; **P<0.01.

To determine the mechanism by which miR-451 regulates cell apoptosis and proliferation in AML cells, we tried to identify the potential targets of miR-451 via several prediction programs and noticed that 3'UTR of *YWHAZ* mRNA contains the sequence motif that could match well with the “seed sequence” of *miR-451* (Figure 5A). The dual-luciferase reporter assay in HEK-293 cells indicated that forced expression of miR-451 significantly suppresses the activity of construct with wild type *YWHAZ* 3'UTR, but not mutant (Figure 5B). Additionally, miR-451 overexpression also inhibited *YWHAZ* expression both at RNA and protein levels (Figure 5C and 5D) in NB4 and HL-60 AML cells.

YWHAZ, also known as 14-3-3-zeta, is a member of 14-3-3 family and could mediate signal transduction by regulating phosphorylation of specific proteins [29]. Here, we found that enforced expression of miR-451 significantly reduced phosphorylated AKT (p-AKT) level, but not total AKT, via targeting *YWHAZ* RNA in the AML cells (Figure 5D). Functionally, knockdown of *YWHAZ* increased cell apoptosis and suppressed cell proliferation (Figure 5E and 5F), suggesting that knockdown of *YWHAZ* could mimic the effects of miR-451 overexpression in the AML cells. Knockdown of *YWHAZ* reduced p-AKT level (Figure 5G, left panel), and knockdown of *c-Myc* suppressed YWHAZ/AKT signaling via activating miR-451 expression (Figure 5G, right panel). c-Myc inhibitor treatment also enhanced cell apoptosis in AML (Figure 5H). Importantly, our rescue assay demonstrated that knockdown of *YWHAZ* could significantly reduce the high level of YWHAZ induced by anti-451 treatment (Figure 5I). Consistent with the YWHAZ expression, the cell apoptosis inhibition by anti-451 could be rescued by re-transfection with si-YWHAZ (Figure 5J). Overall, our data suggested that miR-451 directly targets YWHAZ-AKT signaling to regulating cell apoptosis and proliferation in the AML cells.

### Abnormal overexpression of both c-Myc and YWHAZ is negatively correlated with miR-451 level in AML patients

**Figure 6:**
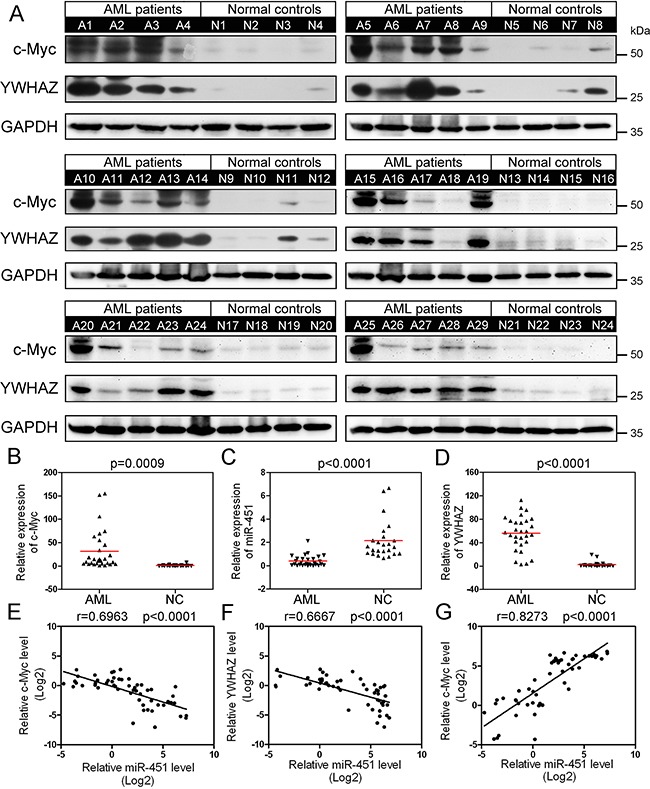
Abnormal upregulation of c-Myc increases expression of YWHAZ via suppressing miR-451 in AML patients **A.** Western blot data indicate that c-Myc and YWHAZ are aberrantly up-regulated in almost all the examined AML patient PB MNC samples. **B-D.** The relative level of c-Myc (B), YWHAZ (C) and miR-451 (D) in the 29 AML patients and 24 healthy donors. The protein levels were quantified using the Image J software and normalized to glyceraldehyde 3-phosphate dehydrogenase (GAPDH). **E-G.** Pearson correlation analysis between c-Myc and miR-451 levels (E), miR-451 and YWHAZ levels (F), as well as c-Myc and YWHAZ levels (G) in the PBMNC samples. The Pearson correlation coefficient r was calculated and verified by the two-tailed significance test.

We randomly selected 29 AML patients and 24 healthy donors, whose peripheral blood samples are available for being used for more analyses, to detect expression of c-Myc and YWHAZ by Western blot (Figure 6A). The results displayed that c-Myc and YWHAZ were abnormally up-regulated in most of the detected AML patients as compared to the normal controls (Figure 6B and 6C), while miR-451 was abnormally down-regulated (Figure 6D). We then analyzed the correlation between c-Myc and miR-451, miR-451 and YWHAZ, as well as c-Myc and YWHAZ. Interestingly, miR-451 level not only negatively correlated with c-Myc expression (Figure 6E), but also YWHAZ (Figure 6F), while expression of YWHAZ was positively correlated with c-Myc expression (Figure 6G) in the examined subjects. We did not observe significant differences of c-Myc and YWHAZ levels among different AML FBA subtypes (data not shown). The results further confirmed the c-Myc⊣miR-451⊣YWHAZ axis in AML.

## DISCUSSION

Abnormal deregulation of miRNAs and the oncogenic or tumor suppressor function of specific miRNAs have been well identified and investigated in tumors [30, 31] as well as in leukemias [32–34], especially in AML [35]. Becker H. et al. and Whitman SP. et al. reported that miR-451 is underexpressed in AML with NPM1 mutation and FLT3-ITD mutation, and deregulation of miR-451 correlated with adverse prognosis in AML with FLT3-ITD mutation [36, 37]. In this study, we find that miR-451 is aberrantly down-regulated in AML patients, and our *in vivo* and *in vitro* data strongly indicates that miR-451 functions as a tumor suppressor through directly increasing cell apoptosis and decreasing cell proliferation in AML.

The proto-oncogene *c-Myc* encodes a transcription factor whose expression is finely regulated during hematopoiesis and frequently hyper-activated in AML [38, 39]. High level of c-Myc functions as a transcriptional amplifier to elevate expression of the already transcripted genes [40, 41]. Strikingly, we identified that hyper-activation of c-Myc results in de-regulation of miR-451 in AML. HDACs regulate gene expression by modifying epigenetic configuration via removing acetyl groups from histone tails, and usually highly expressed in leukemia [14, 42]. Presently, we find c-Myc can recruit HDAC3 on the miR-451 promoter and consequently induce its deregulation in AML. Importantly, knockdown of *c-Myc* or *HDAC3* or treatment with their inhibitors (TSA, HDACs inhibitor; JQ-1, c-Myc inhibitor) results in restoration of miR-451 and mimic its functions in AML. All these findings address that miR-451 repression is a result of c-Myc/HDAC3 interaction in AML.

As critical functional targets of miR-451, we identify that miR-451 directly suppresses *YWHAZ* (14-3-3ζ) in AML. Inhibition of *YWHAZ* could mimic all the functions of miR-451 overexpression. YWHAZ plays a critical role in signaling transduction through interaction with target proteins via phosphorylation motif [43] or directly regulating phosphorylation [44, 45]. We identify that forced expression of miR-451 or knockdown of *YWHAZ* or *c-Myc* result in decreased p-AKT levels in AML. To address whether the c-Myc⊣miR-451⊣YWHAZ regulatory loop is relevant to leukemogenesis, we analyzed the correlation among c-Myc, miR-451 and YWHAZ in AML patients. Consistent our hypothesis, low expression of miR-451 is negatively related with high expression of c-Myc as well as YWHAZ, while c-Myc is positively correlated with YWHAZ level in AML patients.

**Figure 7:**
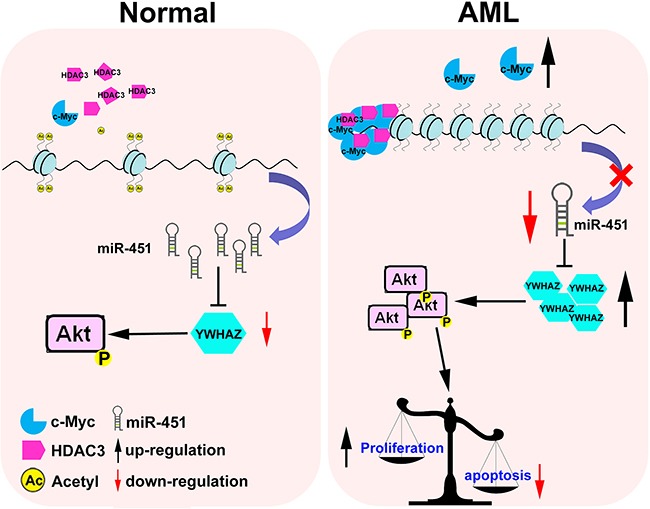
A model for c-Myc/miR-451/YWHAZ/cAKT cascade's being involved in AML development In normal cells, the relative lower level of c-Myc could maintain expression of miR-451 to inhibit oncogenic YWHAZ/AKT signal. While in AML cells, aberrant overexpression of c-Myc could recruit more HDAC3 on the *miR-451* promoter and significantly restrict its expression, which subsequently activates the oncogenic YWHAZ/AKT signal and disturb the balance between proliferation and apoptosis.

Collectively, our study led to the identification of a model for interplay among c-Myc, miR-451, YWHAZ and AKT, as well as their contribution to leukemogenesis (Figure 7). Reintroduction of miR-451 or combination of c-Myc and HDAC inhibitors could be a potential strategy for AML therapy.

## MATERIALS AND METHODS

### Cell lines and their maintenance

The human promyelocytic cell line NB4 and human embryonic kidney cell line HEK-293T were cultured in RPMI-1640 medium (Gibco, BRL, UK) supplemented with 10% FCS (Gibco), 50 U/ml penicillin, and 50 mg/ml streptomycin (Sigma, St. Louis, MO, USA) at 37°C in 5% CO_2_. The human promyelocytic cell line HL-60 was cultured in IMDM medium (Gibco) supplemented with 10% FCS, 50 U/ml penicillin, and 50 mg/ml streptomycin (Sigma) at 37°C in 5% CO_2_. The lentivirus packaging cell line HEK-293TN was maintained in DMEM medium supplemented with 10% FCS, 50 U/ml penicillin, and 50 mg/ml streptomycin (Sigma). HEK-293T cell line was only used for dual-luciferase reporter assay and packaging lentivirus; while NB4 and HL-60 AML cells were used to identify the function of miR-451, YWHAZ, c-Myc and HDAC3.

### Human samples

The PB samples and BM samples from normal volunteers and AML patients were obtained from the 303 Hospital in Nanning, and the 307 Hospital in Beijing, China. The informed consent to perform the biological studies was obtained from all of the examined subjects and the related study was approved by the Ethic Committees of the Institutional Review Board of Institute of Basic Medical Sciences (IBMS), Chinese Academy of Medical Sciences (CAMS). The MNCs were isolated from PB and BM cells by Percoll density gradient [d=1.077] (Amersham Biotech, Germany). The CD34^+^ HSPCs were enriched from BM MNCs through positive immunomagnetic selection (CD34 MicroBead Kit, human, Miltenyi Biotech, Bergisch-Glad-bach, Germany) following the manufacturer's instructions.

### Co-Immunoprecipitation (Co-IP) assay

Co-IP assay were performed as described previously [46]. Antibodies for c-Myc, IgG, HDAC3 and HDAC1 were purchased from Cell Signaling Technology (Danvers, MA, USA).

### Chromatin Immunoprecipitation (ChIP)

2 × 10^7^ NB4 or HL-60 cells for each immunoprecipitation reaction were collected and cross-linked with 1% formaldehyde for 10 min at room temperature. Then chromatin was sonicated to obtain chromatin fragments between 200 and 1000 bp. Immunoprecipitation was performed after overnight incubation with anti-c-Myc or anti-HDAC3 (Cell Signaling Technology) or IgG antibody (Cell Signaling Technology) and subsequent incubation with Protein A agarose (Roche). After reverse cross-linking and DNA purification, the input and the immunoprecipitated DNA samples were used as templates to amplify the target sequences by PCR. The primer sequences are: forward, 5′ CCCTGGGTCCCTATGAGATC 3′; reverse, 5′ CATGGCTTGAAAAGCACTGTG 3′.

### Dual luciferase reporter assay

For *miR-451* promoter activity analysis, a 2703 bp DNA fragment containing the *miR-451* promoter region was amplified using human genomic DNA as template and the primers: forward, 5′ GTCACTTGGGACCTGTCACCTC 3′; reverse, 5′ CTCAGTAATGGTAACGGTTTCCTTG 3′. The fragment containing the wild type and the fragment with deletion of c-Myc binding site, were respectively inserted into pGL3 basic vector (Promega, WI, USA). The c-Myc ORF was amplified using human genomic DNA as template and the primers: forward, 5′- GCCACCATGGATTTTTTTCGGGTAGTG-3′; reverse, 5′-TACATTATGGCTAAATCTTTCAGTCTC-3′. The amplified fragment was inserted into pcDNA3.1. These constructs as well as pRL-TK were transfected into HEK-293T cell together, using Lipofectamine 2000 (Invitrogen, CA, USA). The plasmid pRL-TK containing Renilla luciferase was used as internal control. For miR-451 target analysis, the 3′-UTR of human *YWHAZ* containing the wild type and mutant miR-451 binding site was inserted into pMIR-REPORT. Mutations of the predicted seed regions in *YWHAZ* mRNA sequence were created using the primers including the mutated sequences. HEK-293T cells were co-transfected with 0.4 μg pMIR-REPOTR-YWHAZ construct, 0.02 μg pRL-TK control, and 5 pmol of miR-451 mimic or scrambled controls. Cells were harvested 48 h post-transfection and luciferase activity was assayed with Dual-luciferase reporter assay system according to the manufacturer's protocol (Promega). All transfection assays were carried out in triplicate.

### RNA isolation, reverse transcription (RT) and quantitative PCR (qPCR)

Total RNA was isolated from the harvested cells using TRIzol reagent (Invitrogen, Carlsbad, CA, USA) and quantified by absorbance at 260 nm. cDNA was synthesised by M-MLV reverse transcriptase (Invitrogen) from 0.1–1 μg of total RNA. Stem-poop RT primers were used for the reverse transcription of miR-451 using the primer 5′- GTCGTATCCAGTGCAGGGTCCGAGGTATTCGCACTGGATACGACAACTCAG-3′. U6 snRNA RT primer is 5′-AAAATATGGAACGCTTCACGAATTTG-3′; Oligo18 [10] was used for reverse transcription of mRNAs. q-RT-PCR was carried out in Bio-Rad IQ5 real-time PCR System (Bio-Rad, Foster City, CA, USA) using the SYBR Premix Ex Taq kit (Takara, Dalian, China) according to the manufacturer's instruction. Each assay was performed in triplicate. The data were normalized using the endogenous GAPDH for mRNA and U6 snRNA for miRNAs. The primer sequences used for qRT-PCR were: miR-451 forward, 5′- CTGGAGAAACCGTTACCATTAC-3′; miR-451 reverse, 5′- GTGCAGGGTCCGAGGT-3′; U6 snRNA forward, 5′-CTCGCTTCGGCAGCACATATACT-3′; U6 snRNA reverse, 5′-ACGCTTCACGAATTTGCGTGTC-3′; YWHAZ forward, 5′-TGATCCCCAATGCTTCACAA G-3′; YWHAZ reverse, 5′-GCCAAGTAACGGTAGTA ATCTCC-3′; GAPDH forward, 5′-TCAACGACCACTTT GTCAAGCTCA-3′, GAPDH reverse, 5′-GCTGGTGG TCCAGGGGTCTTACT-3′.

### Cell proliferation and apoptosis

Cell proliferation was determined using the Cell Counting Kit-8 (CCK-8, Dojindo, Kumamoto, Japan) according to the manufacturer's instructions. NB4 and HL-40 cells were seeded into 96-well plates by10000 cells/well. At the indicated time points, 10 μL CCK-8 was added and the cells were incubated for 3 h at 37°C. The optical density was read at 450 nm with a microplate spectrophotometer. Each experiment was performed in triplicate. For apoptosis, the cells were collected, washed once with PBS and resuspended in the 1X binding buffer. Apoptotic cells were assessed using the PE Annexin V Apoptosis Detection Kit 1 (BD Bioscience, San Diego, CA, USA), and immediately analyzed by flow cytometry.

### Western blot

Western blot was performed as described previously [47]. The following antibodies were used. Anti-GAPDH and Anti-YWHAZ were purchased from Proteintech Company (Chicago, IL, USA). Anti-HDAC3, Anti-HDAC1, Anti-p-AKT, Anti-AKT, Anti-c-Myc and Anti-IgG were purchased from Cell Signaling Technology.

### Oligonucleotides, cell transfection and drug treatment

The miR-451 mimics, miR-451 inhibitor, si_YWHAZ, si_c-MYC, si_HDAC3 and their negative controls were purchased from Dharmacon (Austin, TX, USA) and transfected into the AML cells with DharmFECT1 (Dharmacon) at a final concentration of 100 nM. Six hours later, the medium was changed and cells were harvested at indicated time points for analyses. TSA (HDACs inhibitor) and JQ-1 (c-Myc inhibitor) were purchased from Sigma. For apoptosis assay, NB4 AML cells were treated with 300 nM JQ-1.

### Recombination lentivirus production

A 500 bp DNA fragment containing pre-miR-451 was obtained by PCR amplification from human genome DNA with primers: Forward, 5′-CGGAATTCCCCTGGCTGGGATATCATCATATA-3′, Reverse, 5′- TTGCGGCCGCGTATCTATTCCCTCCCCTACCCC-3′. The amplified fragment was inserted into downstream of CMV promoter in pMIRNA1 vector. The lentivirus production and infection are the same as described previously [47].

### Mouse xenograft assay

All experiments involving animals were performed according to protocols approved by the Institutional Animal Care and Use committee of IBMS, CAMS. The mouse xenograft assay was performed as described [47] with some modification. Briefly, 1 × 10^6^ NB4 cells were injected into 4–6 weeks old sublethally irradiated (250cGy) NOD/SCID mice by tail vein and then 1 × 10^7^ lentiviral particles were delivered into the mice once they show typical leukemic symptoms. For mice in the “untreated” group, 100 μL EDTA-PBS were injected. About three weeks later, all the mice were euthanized and the tissues including spleen and BM obtained by femur flushing were collected for further detection. Single cell suspension from spleen and BM was prepared for apoptotic analysis as described above and flow cytometric analysis. Murine spleen tissue was fixed in 10% formalin for 24 h, embedded in paraffin, cut into 4-μm-thick sections and stained with haematoxylin and eosin (H & E).

### Flow cytomety analysis

For detecting engraftment of human NB4 cells into murine BM and spleen, the single cell suspension was washed with chilled PBS and suspended in PBS with 0.5% BSA to block Fc receptors, and were then incubated with PE-conjugated anti-CD33 (BioLegend, San Diego, CA, USA) on ice for 30 min. Finally, cells were washed using cold PBS and fixed in 4% paraform for further analysis on Accuri C6 flow cytometer (BD, SD, USA).

### Statistics

Student's t-test (two-tailed) was performed to analyze the data. The correlations among relative level of c-Myc, miR-451 and YWHAZ were examined by Pearson correlation analysis by GraphPad Prism 5.0. P-values < 0.05 were considered significantly.

## SUPPLEMENTARY MATERIALS FIGURES AND TABLE




