# Supplementary material for: c-Myc suppresses miR-451⊣YWTAZ/AKT axis via recruiting HDAC3 in acute myeloid leukemia
Source: Oncotarget. 2016 Oct 15;7(47):77430–43. doi: 10.18632/oncotarget.12679 (PMC5363596; doi:10.18632/oncotarget.12679)
Supplement: Supplementary file 1 [file oncotarget-07-77430-s001.pdf]

c-Myc suppresses miR-451⊣YWTAZ/AKT axis via recruiting HDAC3 in acute myeloid leukemia

SUPPLEMENTARY FIGURES AND TABLE

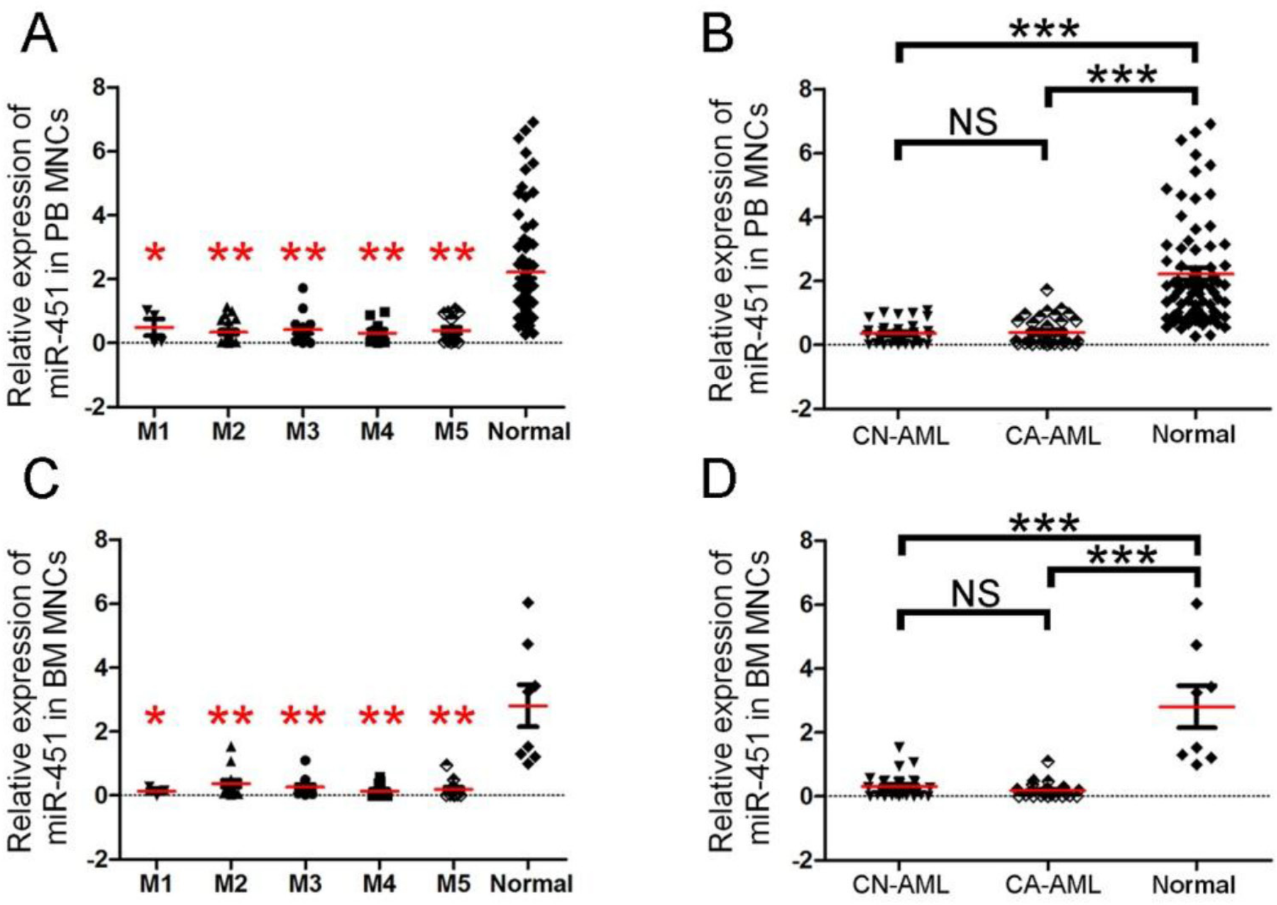

Supplementary Figure S1: Relative expression of miR-451 in different subtypes of AML. A. Relative levels of miR-451 in PB MNCs with different FAB subtypes of AMLs. For M1, n=4; M2, n=25; M3, n=15; M4, n=10; M5, n=15; Normal control, n=80. **P*<0.05; ***P*<0.01 (compared to the normal); Student' test. **B.** Relative levels of miR-451 in PB MNCs from cytogenetically normal AMLs (CN-AMLs) and cytogenetically abnormal AMLs (CA-AMLs). For CN-AMLs, n=34; CN-AMLs, n=35; Normal control, n=80. ****P*<0.001; NS, non-significant. **C.** Relative levels of miR-451 in BM MNCs with different FAB subtypes of AML. M1, n=3; M2, n=15; M3, n=13; M4, n=11; M5, n=13; Normal control, n=9. **P*<0.05; ***P*<0.01 (compared to the normal). **D.** Relative levels of miR-451 in BM MNCs from CN-AMLs and CA-AMLs. For CN-AMLs, n=30; CN-AMLs, n=26; Normal control, n=9. ****P*<0.001; NS, non-significant; The data indicate that deregulation of miR-451 is not specific to FAB subtypes of AML patients or cytogenetically normal or abnormal AMLs.

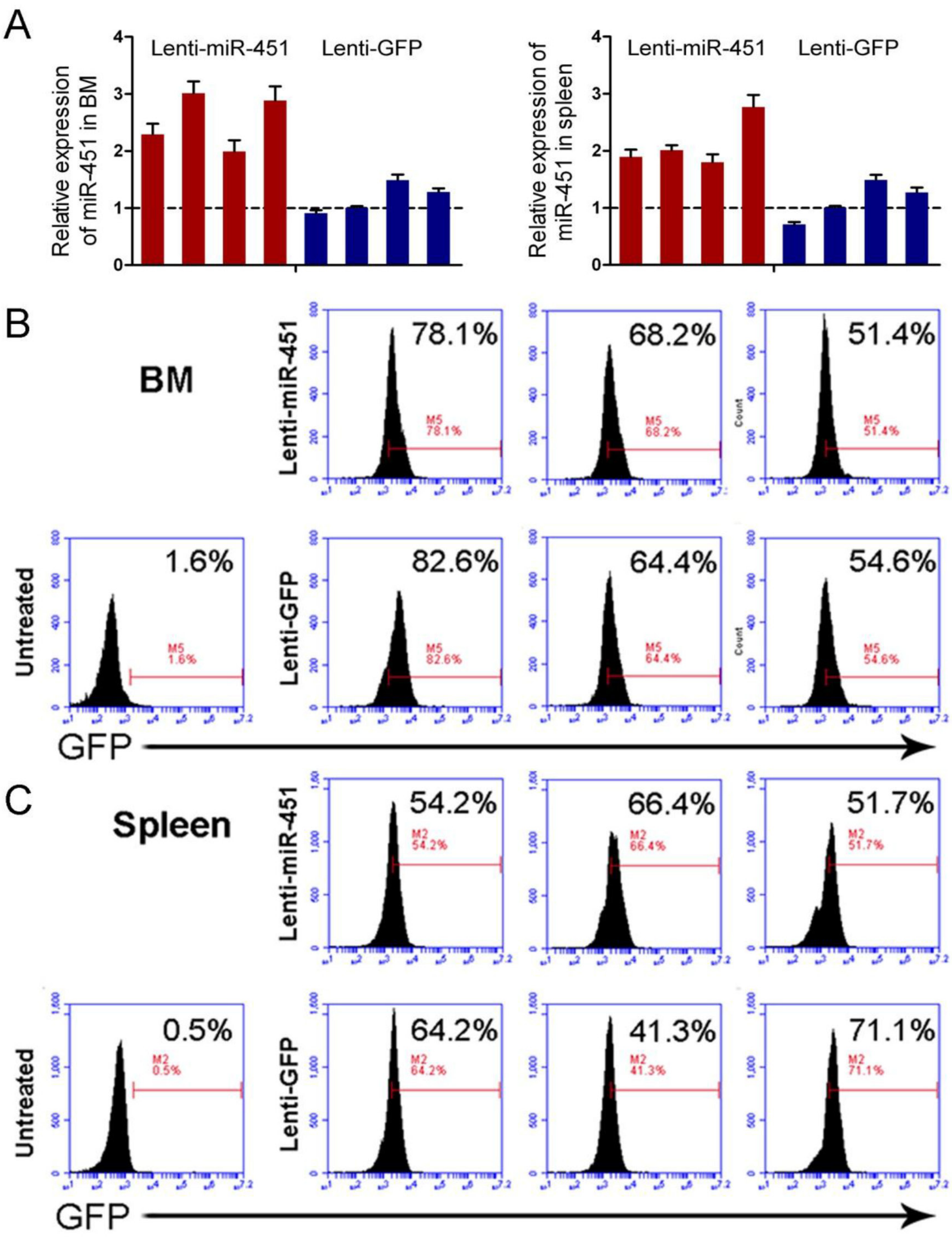

Supplementary Figure S2: Confirmation of the infection efficiency of lentivirus in leukemic mouse model. A. Relative expression of miR-451 in BM and spleen from four lenti-miR-451-injected and four lenti-GFP-injected-mice. **B.** The lentivirus infection efficiency was accessed by GFP levels via FACS analysis in BM. **C.** The lentivirus infection efficiency was accessed by GFP levels via FACS analysis in spleen. Untreated represents the mice without lentivirus injection.

Supplementary Table S1: Characteristics of AML patients used in the detection of miR-451 in PBMNCs, BMMNCs and BM CD34+ HSPCs

See Supplementary File 1
